# Development of a quantitative, portable, and automated fluorescent blue-ray device-based malaria diagnostic equipment with an on-disc SiO_2_ nanofiber filter

**DOI:** 10.1038/s41598-020-63615-2

**Published:** 2020-04-20

**Authors:** Takeki Yamamoto, Muneaki Hashimoto, Kenji Nagatomi, Takahiro Nogami, Yasuyuki Sofue, Takuya Hayashi, Yusuke Ido, Shouki Yatsushiro, Kaori Abe, Kazuaki Kajimoto, Noriko Tamari, Beatrice Awuor, George Sonye, James Kongere, Stephen Munga, Jun Ohashi, Hiroaki Oka, Noboru Minakawa, Masatoshi Kataoka, Toshihiro Mita

**Affiliations:** 1 Department of Tropical Medicine and Parasitology, Faculty of Medicine, Juntendo University, 2-1-1 Hongo, Bunkyo-ku, Tokyo 113-8421 Japan; 2 Panasonic Corporation, Automotive & Industrial Systems Company, Kadoma 1006, Kadoma, Osaka 571-8506 Japan; 3 National Institute of Advanced Industrial Science and Technology (AIST), Health Research Institute, 2217-14 Hayashi-cho, Takamatsu, Kagawa 761-0395 Japan; 4 Nagasaki University, Institute of Tropical Medicine, 1-12-4 Sakamoto, 852-8523 Nagasaki, Japan; 5 Ability to Solve by Knowledge Project, P.O. Box 30, Mbita, Homa Bay 40305 Kenya; 6 Nagasaki University Nairobi Research Station, NUITM-KEMRI Project, P.O. Box 19993, 00202 Nairobi, Kenya; 7 Centre for Global Health Research, Kenya Medical Research Institute, P.O. Box 1578, 40100 Kisumu, Kenya; 8 Department of Biological Sciences, Graduate School of Science, The University of Tokyo, 7-3-1 Hongo, Bunkyo-ku, Tokyo 113-8654 Japan

**Keywords:** Translational research, Malaria, Diagnostic markers

## Abstract

There is an urgent need to develop an automated malaria diagnostic system that can easily and rapidly detect malaria parasites and determine the proportion of malaria-infected erythrocytes in the clinical blood samples. In this study, we developed a quantitative, mobile, and fully automated malaria diagnostic system equipped with an on-disc SiO_2_ nanofiber filter and blue-ray devices. The filter removes the leukocytes and platelets from the blood samples, which interfere with the accurate detection of malaria by the blue-ray devices. We confirmed that the filter, which can be operated automatically by centrifugal force due to the rotation of the disc, achieved a high removal rate of leukocytes (99.7%) and platelets (90.2%) in just 30 s. The automated system exhibited a higher sensitivity (100%) and specificity (92.8%) for detecting *Plasmodium falciparum* from the blood of 274 asymptomatic individuals in Kenya when compared to the common rapid diagnosis test (sensitivity = 98.1% and specificity = 54.8%). This indicated that this system can be a potential alternative to conventional methods used at local health facilities, which lack basic infrastructure.

## Introduction

Globally, malaria is one of the “big three” infectious diseases with an incidence rate of 57 cases per 1000 individuals^1^. Malaria is a vector-borne disease, which is caused by infection from *Plasmodium* spp. and is transmitted by *Anopheles* mosquitoes. The United Nations Sustainable Development Goals had proposed to end the epidemic of malaria by 2030^2^. Although the annual fatality rate has decreased since 2016, about 405,000 malaria-related deaths were reported in 2018. Similarly, new malaria cases have increased slightly since 2014 with 231 million recorded cases in 2017 and 228 million recorded cases in 2018^3^. Several factors have stagnated global progress in eradicating malaria^4^. Particularly, most tools to tackle the current malaria infection were developed before 2000. Thus, there is a need to develop new tools using novel technologies to accelerate the efforts toward malaria elimination^4^.

Accurate diagnosis is essential to ensure effective patient management and to prevent unnecessary treatment. Rapid diagnostic tests (RDTs), which detect malaria-specific antigens, such as histidine-rich protein 2 (HRP2), are easy to use, rapid, and affordable. Hence, RDTs are widely used even in remote areas. However, RDTs are unable to determine the quantitative content of parasites in the blood (parasitaemia) that is generally associated with the severity of malaria infection. Additionally, as the clearance time of HRP2 antigen in the patient’s blood is very long, the presence of residual antigen potentially produces a persistent false-positive result even after several weeks post-parasite clearance^5^. Furthermore, the deletion of the *HRP2* gene results in a false-negative result^6^. The microscopic examination of Giemsa-stained blood smear, which is another common diagnostic method, is a low-cost method for the determination of parasitaemia. However, microscopic examination involves labour-intensive steps and requires technical expertise for an accurate diagnosis.

Recently, we had developed a highly sensitive and rapid malaria diagnosis system using fluorescent blue-ray optical devices^7^. The system comprises a scan disc and a fluorescence image reader. The erythrocytes are dispersed onto the disc surface where malaria parasites are fluorescently stained. The fluorescence-positive erythrocytes are then detected by the fluorescence image reader. There was a linear correlation between our malaria diagnostic system and microscopic examination for the detection of parasitaemia. However, further improvements are needed to render the current system a fully automated malaria diagnosis equipment that can be used in malaria-endemic area. Previously, the samples that contained only erythrocytes (a standard laboratory-cultured *P. falciparum*-infected and non-infected erythrocytes) were used. However, the patient blood samples also contain leukocytes and platelets, which potentially produce fluorescent noises. These noises interfere with the accurate detection of malaria. In some cases, these noises are incorrectly recognised as malaria parasites. Additionally, *P. falciparum* strains in the patient blood in malaria-endemic regions are highly diverse. The previous study was performed in a well-equipped laboratory. The laboratory conditions are different from the conditions in the malaria-endemic regions, which have limited infrastructure. The current system needs a centrifugation step to remove the leukocytes and platelets, which usually requires huge electric power and several complicated manual steps before applying blood samples into the system.

In this study, we first developed a SiO_2_ nanofiber filter device and incorporated it into the scan disc, which can be operated automatically without the need for centrifugation. This modification enabled the conversion of the current system into a fully automated malaria diagnosis equipment. We then validated the applicability of the newly developed equipment for the diagnosis of *P. falciparum* in individuals from the malaria-endemic region of Kenya.

## Results

### Design of automated malaria diagnostic system

**Figure 1:**
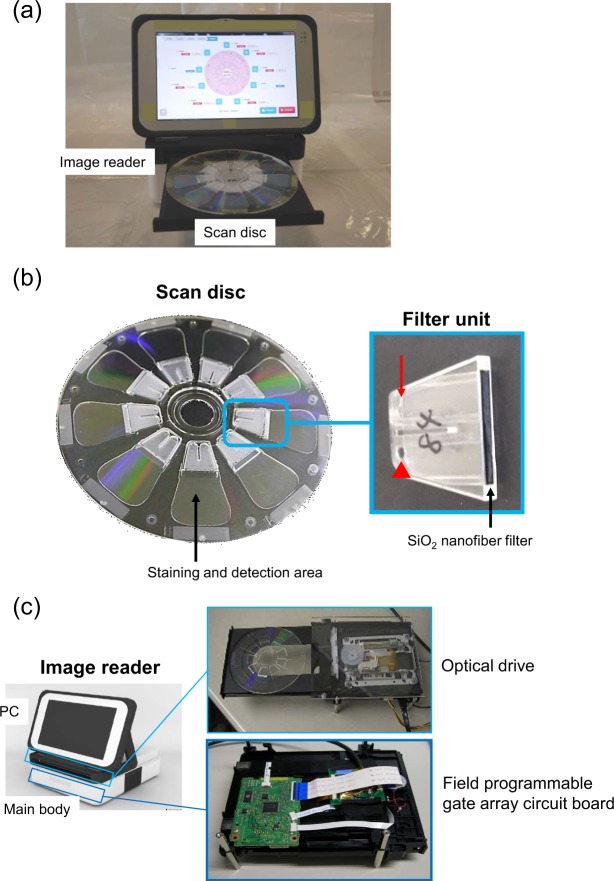
Design of the fully automated, quantitative malaria diagnostic system. (**a**) The system consists of an image reader (upper) and a scan disc (lower). (**b**) Overall view of the scan disc (left) and the filter unit (right). The filter unit has an air vent (arrow) and a sample injection port (arrowhead). The schematic diagram of a cross-section of the scan disc and details of the filter unit design are shown in Supplementary Fig. S2. (**c**) Fluorescence image reader consists of a tablet PC (upper) and a main body (lower) equipped with a Blue-ray optical component.

The automated malaria diagnostic system is primarily composed of the following two devices (Fig. 1a): scan disc (EZBNPC02AT, Panasonic Corp., Osaka, Japan) (Fig. 1b) and fluorescence image reader (EZBLMOH02T, Panasonic Corp.) (Fig. 1c). The scan disc with the same 120 mm diameter as CDs and DVDs has a flow-path disc component and an optical disc component with a *Plasmodium* staining unit. The function of the scan disc is to isolate the erythrocytes and deploy them in a monolayer formation onto the staining unit. In the staining unit, the malaria parasites are fluorescently stained with a nuclear-specific fluorescence stain, Hoechst 34580 (Molecular Probes Inc., Eugene, OR, USA). The fluorescence image reader detects the fluorescently stained nuclei of the malaria parasites. The identification of *P. falciparum* and the quantitative measurement of the proportion of infected erythrocytes among all counted erythrocytes are performed using custom-made software.

### Development of on-scan disc SiO_2_ nanofiber filter unit

We previously reported a push-column nanofiber filter, which removes leukocytes and platelets from the blood samples^8^. Based on this, we developed a scan disc SiO_2_ nanofiber filter unit. We strictly controlled the pore size and the time required for the formation of the SiO_2_ nanofiber (Supplementary Fig. S1a). This modification enables nearly all leukocytes and platelets to be trapped in the nanofiber and only the erythrocytes to pass through this filtration system (Supplementary Fig. S1b). As the SiO_2_ nanofiber filter unit is an enclosed space (Fig. 1b), an air vent is indispensable to inject a 200-µL volume of the diluted blood sample. We placed the sample injection port and the air vent hole on the inner peripheral side and created a partition in the middle of the SiO_2_ nanofiber filter unit (Supplementary Fig. S2). This prevented sample leakage from the hole located on the outer periphery during sample centrifugation. The centrifugal conditions were also optimised at 1000 rpm for 30 s (Supplementary Fig. S3). We finally confirmed that the erythrocytes were evenly distributed on the detection area without any bubble formation inside the disc. The development of on-scan disc SiO_2_ nanofiber filter unit renders the previous system to be a fully automated malaria diagnostic apparatus.

**Figure 2:**
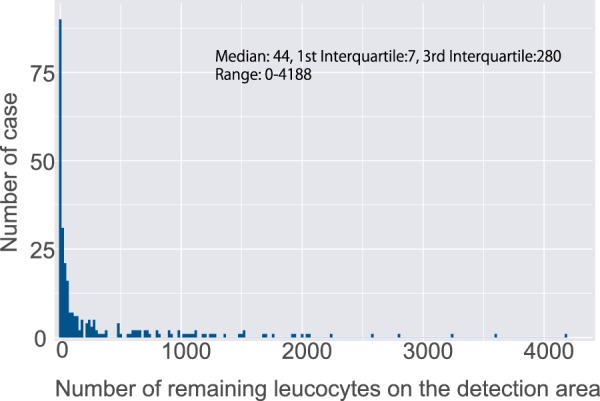
Evaluation of an on-disc SiO_2_ nanofiber device for the removal of leukocytes. Remaining leukocytes on the detection area. The median number of remaining leukocytes was 44 (7 in the first quartile, 280 in the third quartile). Blood samples from Kenyan individuals were used (n = 274) for the analysis.

We verified the performance of on-scan disc SiO_2_ nanofiber filter unit using the blood samples obtained from Japanese volunteers. The results revealed that the mean and median removal percentages of leukocytes were 99.7% and 100%, whereas those of platelets were 90.2% and 91.7%, respectively (Table 1). We also confirmed that the number of leukocytes remaining on the detection area after SiO_2_ nanofiber filtration was low (median 44) in 274 Kenyan blood samples (Fig. 2, Table 2). These results indicated that the developed filtration system can remove most of the leukocytes from the blood samples obtained from the individuals living in malaria-endemic region.

**Table 1:** Evaluation of reformed SiO_2_ nanofiber device for the removal of leukocytes and platelets in healthy Japanese volunteers.

	Leukocyte removal rate (%)	Platelet removal rate (%)
Number of samples^a^	11	11
Average	99.7	90.2
Median (1st quartile, 3rd quartile)	100 (100, 100)	91.7 (83.3, 100)

^a^blood samples of healthy Japanese volunteers.

**Table 2:** Removed leukocytes on the detection area in 274 Kenyan individuals.

Remaining leucocytes on the detection area	N
0–19	108
20–199	83
200–1000	52
1000-	31

### Limit of detection (LOD) of the 18S rRNA nested polymerase chain reaction (nPCR)

The 18S rRNA was analysed by nPCR to detect the malaria parasites. The LOD of the parasite density was determined by nPCR using a laboratory-adapted 3D7 clone at a density range of 0.0375 to 4 parasites/μL (using 2-fold dilutions in 12 different rows) (Supplementary Fig. S4). Probit analysis was performed to determine the density at which the parasite could be detected with 95% confidence. The analysis revealed that the LOD of the nPCR was 2.56 parasites/μL.

### Study subjects and malaria positive rates

Among the 288 school children enrolled in this study, 11 were excluded as we did not obtain an adequate amount of blood samples (n = 3) or because the blood coagulated (n = 8) (Supplementary Fig. S5). The nPCR analysis was performed on the samples from the remaining 277 individuals, which revealed that 56 (20.2%) samples tested positive for the malaria parasite. Of these, 48 tested positive only for *P. falciparum*, 4 samples tested positive for *P. falciparum* and *P. ovale*, 1 tested positive for *P. falciparum* and *P. malariae*, and 3 tested positive only for *P. ovale* (Table 3). As this study focused on the diagnosis of *P. falciparum*, we excluded three samples that tested positive only for *P. ovale* from further analysis. In total, 274 individuals (95.1% of the total enrolled subjects) underwent a diagnosis test for malaria parasites.

**Table 3:** Clinical characteristics of 274 Kenyan individuals enrolled in the study.

Characteristics	
**Age (years)**	
≤2	29
3–5	60
6–10	93
11≤	92
Average	8.4
**Sex**	
Male	119
Female	155
**Hemoglobin (g/dL)**	
<7	3
7–9	22
10–13	162
13<	87
Average (95% CI)	12.1 ± 0.2
***Plasmodium*** **infection status evaluated by nested PCR (number of individuals)**	
*Pf*	48
*Pf+Po*	4
*Pf+Pm*	1
Negative	221
Parasitemia; Median (range)	0.04% (0.00043%–1.32%)
***Plasmodium*** **infection status evaluated by RDT**^**a**^ **(number of individuals)**	
Positive	152
Negative	122

^a^Rapid diagnostic test.

The mean age of the participants was 8.4 years (Table 3). The mean haemoglobin level among the study subjects was 12.2 g/dL. Only three individuals had a haemoglobin level of less than 7 g/dL. The median percentage of microscopic measured parasitaemia in the nPCR-determined malaria parasite-positive samples was 0.04% (range: 0.00043–1.3%). The sensitivity and specificity of the RDT were 98.1% and 54.8%, respectively (Table 4). The negative predictive value of the RDT was very high (99.2%), whereas the positive predictive value was low (34.2%), which were similar to those previously reported in Kenya^9^ and Democratic Republic of Congo^10^.

**Table 4:** Diagnostic performance of *Plasmodium falciparum* infections in 274 Kenyan individuals.

	Sensitivity	Specificity	PPV^a^	NPV^b^	Accuracy
Malaria diagnostic system	100.0%	92.8%	76.8%	100.0%	94.2%
	(93.3%–100%)^c^	(88.5%–95.8%)	(65.1%–86.1%)	(98.2%–100%)	(90.7%–96.6%)
RDT^d^	98.1%	54.8%	34.2%	99.2%	63.1%
	(90.0%–100%)	(47.9%–61.4%)	(26.7%–42.3%)	(95.5%–100%)	(57.1%–68.9%)

^a^Positive predictive value, ^b^Negative predictive value, ^c^95% confidence interval, ^d^Rapid diagnostic test.

### Determination of parasitaemia using the automated malaria diagnostic system

**Figure 3:**
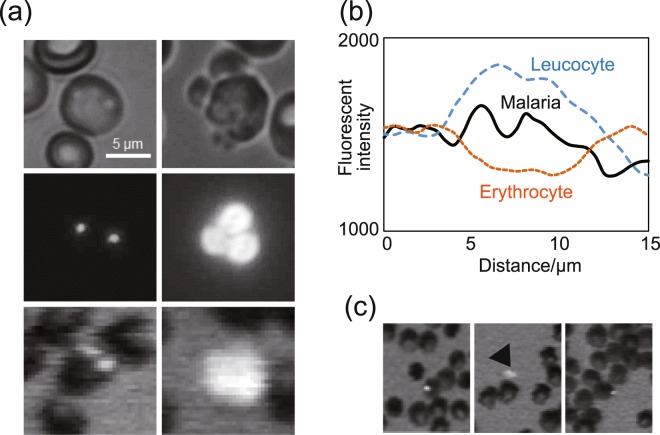
Discrimination of malaria parasites, leukocytes, and platelets on the fluorescent blue-ray optical system. (**a**) Malaria parasite (Left) and leukocytes dispersed (Right) on the scan disc. Differential interference-contrast microscopic images (Upper), conventional fluorescence microscopic images (Middle), and fluorescence images captured by the fluorescent blue-ray image reader (Lower). (**b**) Fluorescence intensity profile of erythrocyte with the malaria parasite (Black line), uninfected erythrocyte (Orange dotted line), and leukocytes (Blue dotted line). The fluorescence intensity was measured along the yellow arrow in each image in Supplementary Fig. S8. (**c**) Fluorescence images of malaria parasite (Left), platelet (Middle, arrowhead) and platelet on the erythrocyte (false-positive) analysed on the fluorescent blue-ray image reader.

After the removal of leukocytes and platelets, the erythrocytes were allowed to spread on the detection area and were stained with a pre-adsorbed nuclear-specific fluorescence dye (Hoechst 34580). The staining yielded fluorescent-positive images of the malaria parasites in the infected cells (Fig. 3a). Erythrocytes that include a fluorescent spot with an area from 1.0 μm^2^ to 10 μm^2^ were considered to be malaria infected erythrocytes. The fluorescence intensity of the malaria parasite was markedly lower than that of leukocytes (Fig. 3b, Supplementary Fig. S6). As haemoglobin has a strong absorption peak at the excitation wavelength of 400 nm^11^, erythrocytes can be visualised by the image reader in our diagnostic system, which enables us to visually count the number of erythrocytes. Therefore, the proportion of infected erythrocytes among the total counted erythrocytes can be measured with the fluorescent image reader software. Although platelets are generally not stained by Hoechst 34580, we observed that a very small proportion of platelets was stained with this dye. In most cases, these platelets were located outside the erythrocyte with distinguishable shape and fluorescent intensity when compared to the malaria parasites (Fig. 3c).

The average number of purified erythrocytes on the detection area was 836,863 (95% confidence interval (CI): 737,174–936,552) in the *P. falciparum*-positive samples (n = 53) and 912,556 (95% CI: 862,220–962,893) in the *P. falciparum*-negative samples (n = 221) (Supplementary Table S1). The number of fluorescent-positive spots diagnosed as malaria parasites ranged from 23–5,130 in the *P. falciparum*-positive samples. However, 16.1 spots per sample were also incorrectly recognised as malaria parasites on an average in the *P. falciparum*-negative samples.

We first determined the critical value (CV) of our diagnostic system, which is defined as the value that produces an error probability of 0.05 when parasite true negative samples are measured. We used the Currie method that is recommended by the International Union of Pure and Applied Chemistry and commonly used for this purpose^12,13^. The CV was determined as 0.0048% based on the mean (0.0018%) and standard deviation (SD) (0.0018%) of the percentage of parasites determined by our diagnostic system in parasite true negative samples (n = 221) (Supplementary Table S2). As the CV is generally used as a cut-off value for distinguishing positive results from negative results^12–14^, we adopted 0.0048% (216 parasites/µL) as the cut-off value for detecting parasitaemia using our diagnostic system.

The sensitivity and specificity of the malaria diagnostic system were 100% (95%CI, 93.3–100%) and 92.8% (95%CI, 88.5–95.8%), respectively (Table 4). The positive and negative predictive values were 76.8% (95%CI, 65.1–86.1%) and 100% (95%CI, 98.2–100%), respectively. When these values were stratified by parasitemia, specificity was 92.8% (95%CI, 88.5–95.8%) in parasite negative samples; 100% of sensitivity was observed in the group of low parasitemia (0–0.01%) (Table 5). The specificity obtained by our diagnostic system was significantly higher than that obtained by RDT (54.8%) (P = 2.2×10^–16^, McNemar’s test). To assess the variability in diagnostic accuracy across different groups of participants, we analysed the blood samples obtained from Japanese volunteers (n = 40) (Supplementary Table S3). The malaria parasite-positive samples were prepared by adding the *P. falciparum* laboratory clone (3D7) to the blood samples and the samples were analysed using our diagnostic system. The sensitivity and specificity of the malaria diagnostic system were 93.3% (95%CI, 68.1 to 99.8) and 92.0% (95%CI, 74.0 to 99.0), respectively. This indicated that the diagnostic values obtained in the blood samples of Japanese individuals were similar to those obtained in the blood samples of Kenyan individuals.

**Table 5:** Diagnostic performance of malaria diagnostic system by parasitemias in 274 Kenyan individuals.

	N	Sensitivity	Specificity
Parasite negative	221	ND^a^	92.8% (88.5%–95.8%)^b^
0 < parasitemia ≤ 0.01%	18	100%	ND
0.01% < parasitemia ≤ 0.1%	16	100%	ND
0.1% < parasitemia	9	100%	ND

^a^ND, ^b^95% confidence interval.

We also determined the LOD, which is defined as the value that produces an error probability of 0.05 when samples having a LOD level are measured according to the Currie method^12,13^. The calculated LOD was 0.0077% (347 parasites/µL) (Supplementary Table S2).

### Regression analysis of parasitaemia determined by automated malaria diagnostic system and microscopy

We evaluated the degree of correlation between the percentage parasitaemia obtained by our diagnostic system and that obtained by microscopy in 53 malaria parasite-positive samples (Supplementary Table S4). As the percentage parasitaemia values did not exhibit a normal distribution in both methods, the data were log-transformed. Pearson’s correlation test revealed a significant correlation between the parasitaemia percentage values determined by the two methods (r = 0.80, P = 1.2 × 10^−12^). A strong correlation was also obtained in Spearman’s rank-correlation test (r = 0.83, P = 2.6 × 10^−29^). These results indicated a linear correlation between the percentage parasitaemia value obtained by the automated malaria diagnostic system and that obtained by microscopy.

Next, we performed a linear regression analysis to correlate the percentage parasitaemia values obtained by the two detection methods (Supplementary Fig. S7). This analysis yielded the following equation: Predicted logarithmic transformed parasitaemia percentage evaluated by microscopy $=\,0.74+(1.40\times$ parasitaemia percentage evaluated by automated malaria diagnostic system) with an adjusted R^2^ value of 0.6254 (P = 1.13 × 10^−12^). As mentioned previously, the CV and LOD for our diagnostic system were 0.0048% and 0.0077%, respectively (Supplementary Table S2). Using this regression equation, the corresponding CV and LOD for the microscopically determined parasitaemia were estimated to be 0.0031% (140 parasites/µL) and 0.0061% (275 parasites/µL), respectively (Supplementary Table S2).

## Discussion

Previously, we had performed an *in vitro* evaluation of our fluorescent blue-ray optical device-based malaria diagnostic system^7^. In this study, we developed an on-disc SiO_2_ nanofiber filter device that can automatically remove leukocytes and platelets, which are the major inhibitory factors for accurate malaria diagnosis in our system, from the blood samples using centrifugal force. This device has rendered the malaria diagnostic system a fully automated quantitative apparatus. The only manual step involved in the use of this system is the dilution of the blood sample. We next evaluated the field application of the new apparatus in Kenya. For this study, we enrolled Kenyan individuals who exhibited a very low parasitaemia percentage (median: 0.04%, range: 0.00043–1.3%). The automated malaria diagnostic system could detect malaria parasites with a high sensitivity value (100%). Furthermore, the specificity (92.8%) of our diagnostic system was markedly higher than that of other commercial RDTs (55%). The high specificity of diagnosis may prevent unnecessary administration of antimalarial drugs to non-malaria parasite-infected individuals. Consequently, this may alleviate the risks associated with the emergence and spread of drug-resistant malaria parasites^15^.

The estimated CV and LOD of our diagnostic system in Kenyan individuals were 0.0031% (140 parasites/µL) and 0.0061% (275 parasites/μL), respectively, which are comparable to the LOD of the microscopic examination with Giemsa staining by a skilled microscopist and RDT. The factor that negatively affects the CV and LOD is platelets, which could not be removed in the system designed in our previous study. Further improvement of the filtration performance can potentially decrease the number of platelets in the detection area. Platelet aggregation using collagen activation is a potential strategy because it is inexpensive and simple^16^. Further improvement of imaging resolution in the detection area can also improve the ability of the system to distinguish the malaria parasites from the accidentally stained platelets. Although a resolution of 500 nm has been already achieved, a higher resolution can be achieved using super-resolution techniques, which include fine measurements with a smaller spot size and an objective lens having a higher numerical aperture and/or digital reconstruction of fine images by image convolution^17,18^. When the blood samples that were pre-treated for erythrocyte transfusion were analysed, the platelets were almost completely removed and the LOD for detecting the parasite was 0.0002% (9 parasites/μL)^7^. Therefore, we believe that a similar LOD level can be achieved using the above-mentioned improvements.

The automated diagnostic system can determine parasitaemia, which provides a comparative advantage over RDT that has been used as a point of care testing (POCT). The determination of parasitaemia enables the evaluation of therapeutic efficacy by monitoring the patient’s parasitaemia status after treatment and the early detection of antimalarial-resistant cases. It also provides clinical benefit for patients with severe malaria because high parasitaemia is one of the important manifestations of suspected severe malaria. Therefore, it is reasonable to develop a novel malaria diagnostic system that can determine parasitaemia. Various new technologies have been used for this purpose, including magnetic resonance relaxation^19^, flow cytometry^20^, automatic counting from digitally captured images of Giemsa-stained blood smears^21,22^, and the evaluation of acoustic signals of vapour-generated nanobubbles from hemozoin^23^. Although some technologies are highly sensitive for the detection of malaria parasites with minimal blood volume, they might not be ideal for POCT because of the size of systems and/or requirement of huge power supply, which are generally limited in the remote areas with poor infrastructure.

Contrastingly, the automated malaria diagnostic system is portable, battery-driven, and robust. These characteristics are important as malaria diagnosis is commonly performed in tropical regions with severe conditions, such as high temperature, humidity, and dusty environment. The system is designed for protection against particles and water based on the criteria stipulated by the International Electrotechnical Commission IP52 (IEC 60529, “Degrees of protection provided by enclosures (IP Code),” 2013). We have not observed the errors caused by heat, humidity, or dust after repeated experiments with the automated diagnostic system in the field. The scan discs were also stable for several months at a wide temperature range (10–35 °C)^7^. However, we further improved the packaging using an aluminium bag. Additionally, the inert N_2_ is cycle purged, which prevents humidity and oxygen permeation that may degrade the fluorescent dyes. Furthermore, the automated malaria diagnostic system is easy to operate and enables the analysis of stable parasitaemia independent of technical expertise. The scan discs can be manufactured at low-cost because a nanofiber filter chip inside the scan disc is manufactured by mass-production using a wafer. The cost of 1 $/sample can be realised even if necessary components, such as pipette tips and phosphate-buffered saline (PBS) are included. The image reader can be used on any tablet or laptop PC by installing the software, and the main body is expected to be priced at similar levels to commercial blue-ray players.

In conclusion, we have developed an automated and quantitative malaria diagnostic system. Field testing of the system in Kenya revealed that the diagnostic system has high diagnostic accuracy. Compared to the Giemsa staining method, the automated system is associated with shorter diagnosis time, minimal human errors, higher specificity, and the ability to quantitatively measure parasitaemia. These results indicate the potential of this diagnostic system as a valid alternative to conventional methods used at local health facilities, which lack basic infrastructure. However, further studies using a large sample size are needed to verify the applicability of the automated malaria diagnostic system.

## Methods

### Fabrication of SiO_2_ nanofiber filter chip and filter unit

**Figure 4:**
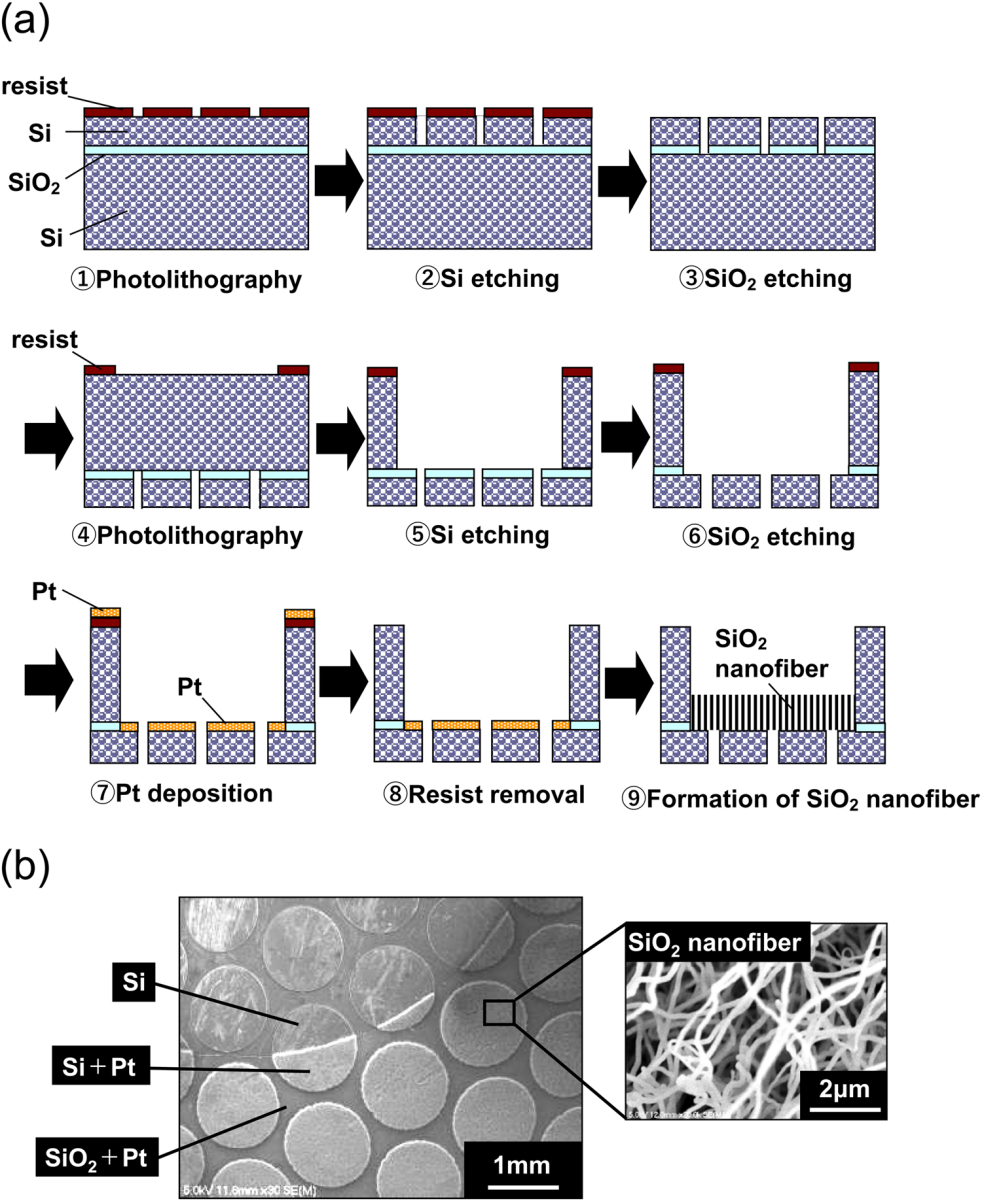
Fabrication of SiO_2_ nanofiber filter chip. (**a**) Process flow of SiO_2_ nanofiber filter chip with cavity structure. (**b**) Fine patterning of SiO_2_ nanofiber (Both Si substrate and platinum catalyst are required for SiO_2_ nanofiber formation).

SiO_2_ nanofiber chips were made from silicon wafer using the micro-electromechanical system (MEMS) processing technology, which involves lithography and etching (Fig. 4a). The SiO_2_ nanofiber was allowed to form only on the inside of the Si chip using the Vapour-Liquid-Solid (VLS) method^24,25^. The VLS method is a vapour phase growth method in which SiO_2_ vapour is melted into liquid metal (platinum) particles and the nano solid phase (SiO_2_) precipitates when SiO_2_ reaches supersaturation^26^. SiO_2_ nanofiber is produced only in the presence of Si and platinum catalyst^26^. Hence, it is possible to form SiO_2_ nanofiber only at an arbitrary position by patterning platinum catalyst or patterning SiO_2_ (Fig. 4b). SiO_2_ has been selected as a cell adhesive material that promotes the removal of leukocytes and platelets, which enables the maintenance of small dead volume and efficient removal. Additionally, SiO_2_ nanofiber is directly bonded to the base material. In contrast to the nanofiber sheet produced by the melt blow method, the SiO_2_ nanofiber formed inside the Si chips by the VLS method is not fragile and has excellent mounting workability.

Next, we developed a SiO_2_ nanofiber filter unit that was small enough (15 mm × 2 mm, 250 µm thickness) to be placed inside the scan disc (Fig. 1a,b). There are two holes on the surface of the filter unit: a sample injection port for injecting the sample with a pipette and an air vent hole for filling the inside of the filter unit with the sample. The case for fixing the filter is a plastic product made by general injection moulding, and the filter chip was fixed using an ultraviolet (UV) curable adhesive. The SiO_2_ nanofiber filter unit was positioned next to the blood sample detection area. When the blood sample is applied to the scan disc, it first passes through the SiO_2_ nanofiber filter, where the leukocytes and platelets are trapped, resulting in effective erythrocyte isolation.

### Malaria diagnosis protocol by the automated malaria diagnostic system

**Figure 5:**
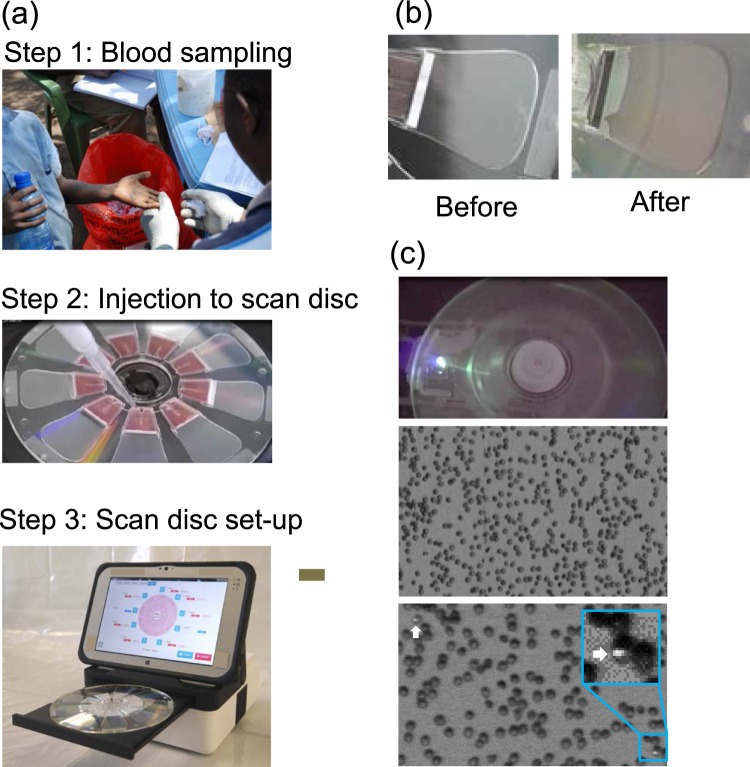
Process for malaria diagnosis using the automated malaria diagnostic system. (**a**) The manual steps involved in the automatic malaria diagnostic system: blood sampling, injection into the scan disc, and scan disc setup. (**b**) Diluted samples before and after filtration by centrifugal force. (**c**) Fluorescent images of erythrocytes in the detection area captured by the automated malaria diagnostic system. (Top) Acquisition of fluorescent images (Middle) erythrocytes are deployed in a monolayer formation. (Bottom) Malaria parasites (arrows) are fluorescently stained in the detection area. High-magnification fluorescent image of *Plasmodium falciparum*-infected erythrocytes on the disc. The target malaria parasites were analysed quantitatively at the single-cell level.

The quantitative malaria diagnosis of the automated malaria diagnostic system comprises six steps as follows: Step 1, the finger-prick blood sample is collected into a capillary tube. The blood (2 µL) is then manually diluted using PBS as a buffer solution (198 µL); Step 2, the diluted blood sample is injected into the scan disc; Step 3, the scan disc is set up in the fluorescence image reader; Step 4, the diluted sample is automatically passed through the SiO_2_ nanofiber filter by centrifugal force (Fig. 5a). The purified erythrocytes are deployed onto the detection area in a monolayer formation (Fig. 5b); Step 5, malaria parasites present in the sample are fluorescently stained and the fluorescent signals are captured by the fluorescence image reader (Fig. 5c); Step 6, the number of erythrocytes and malaria parasites present in the erythrocytes is automatically estimated from the fluorescent image by the built-in image-processing software. The scan disc can analyse nine samples simultaneously in approximately 40 min. The time required for the measurement is proportional to the scanning distance of the radial direction of the scan disc. If the number of erythrocytes to be measured can be reduced, it is possible to shorten the measurement time.

### Study site for the evaluation of field applicability and diagnostic performance of the developed system

A field test was conducted between 14 and 23 February 2018 to evaluate the performance and field application of our diagnosis system. The area of the study region, which is in the Gembe East Sub-location in Homa Bay County (Mbita District, Nyanza Province, western Kenya, 0°28′24.06′′S, 34°19′16.82′′E), was approximately 12 km^2^ and included 14 villages^27^. In this site, the annual rainfall ranges from 700 to 1200 mm with two rainy seasons (from March to June and from November to December). All *Plasmodium* species that cause malaria in humans, except *Plasmodium vivax*, were reported in this region. Additionally, *P. falciparum* (>90%) was reported to be the most prevalent species in this region^28^. The prevalence rate of malaria parasites in the study region evaluated by microscopic diagnosis and PCR was approximately 15–24% and 30–44%, respectively^28^. *Anopheles gambiae sensu stricto*, *Anopheles arabiensis*, and *Anopheles funestus* are the main malaria vectors in the study region^29,30^.

### Blood collection

The minimum number of subjects to be enrolled for the study was determined based on the table of power estimates reported by Flahault *et al*.^31^. According to their study, 50 infected individuals are sufficient to detect *P. falciparum* with an expected sensitivity of 95% and a lower bound 95% CI value of 80%. We assumed that the prevalence of *P. falciparum* infection in the study area was 20%, which estimated that 250 individuals must be recruited for the study. This estimate is well matched to the sample size used in our study.

The study was conducted using a community-based cross-sectional survey in all villages and 14 primary/secondary schools. The blood samples were collected from school children aged 1-16 years using a finger-prick technique and stored in BD Microtainer Tubes containing K_2_EDTA (Becton, Dickinson and Company, Franklin Lakes, NJ, USA). We analysed a maximum of 40 samples in one day at the central laboratories of the International Centre of Insect Physiology and Ecology (Mbita, Kenya). The haemoglobin level was measured using a HemoCue Hb201+ system (HemoCue AB, Ängelholm, Sweden). *P. falciparum* infection was screened using a commercial RDT kit (Paracheck-*Pf*® Rapid Test for *P. falciparum*, ver. 3, Orchid Biomedical Systems, Verna, Goa, India). We prepared thin and thick blood smears on site. All smears were stained with 10% Giemsa solution for 10 min and examined under oil immersion objected of a light microscope (Olympus, Co., Ltd., Tokyo, Japan) at 1000X magnification. For molecular analysis, whole-blood samples (20 µL) were transferred onto Whatman FTA® microcards (GE Healthcare, Chicago, IL, USA). The samples were allowed to dry at room temperature and stored separately in plastic bags at −20 °C. DNA was extracted from the 5.5-mm-diameter blood spots using the QIAamp DNA Micro Kit (QIAGEN, Venlo, Netherlands). The final elution volume was 20 µL.

The purpose and procedure of the study were informed to the participants through local interpreters. Written informed consent was obtained from their parents or legal guardians. Individuals who tested positive for malaria were treated with Artemether–lumefantrine. We obtained ethical approval to conduct the human study from the Kenya Medical Research Institute Ethical Review Committee (KEMRI/RES/7/3/1, SSC No. 3168), and the National Institute of Advanced Industrial Science and Technology (AIST) ethics committee (No. 2017-156). All experiments were performed in accordance with Ethical Guidelines for Medical and Health Research Involving Human Subjects stipulated by the Ministry of Education, Culture, Sports, Science and Technology and the Ministry of Health, Labour and Welfare (revised on February 28, 2017).

### Diagnosis of malaria by 18S rRNA nPCR and microscopic examination

Species-specific nPCR was used to detect the *P. falciparum* infection as described previously^32,33^. This method targets the 18S rRNA gene of *P. falciparum*^32,33^. The genome of *P. falciparum* has 5–8 copies of 18S rRNA. The 18S rRNA is commonly used for DNA-based malaria detection methods^34^. The reported LOD of this method varies from 1 to 50 parasites/μL^32,35–37^. In this study, we determined the LOD of parasite density using a laboratory-adapted 3D7 clone at a density range of 0.0375–4 parasites/μL (using 2-fold dilutions in 12 different rows). Probit analysis was performed to determine the minimal density at which the parasite would be detected with 95% confidence. Parasitaemia in *P. falciparum*-positive cases was determined by counting 10,000 erythrocyte using thin blood smears or counting 500 leukocytes using thick blood smears.

### Statistical analysis

To estimate the CV and LOD, we used the Currie method that is recommended by the International Union of Pure and Applied Chemistry and commonly used for this purpose^12,13^. The CV, which is defined as the value that produces an error probability of 0.05 when true negative samples are measured, was used as the cut-off level to distinguish the parasite-positive cases from the parasite-negative cases by this system. The CV was determined based on the following formula: CV = mean + 1.645 SD. The LOD, which is defined as the value that produces an error probability of 0.05 when samples having a LOD level are measured, was calculated using the following formula: LOD = mean + 3.29 SD^12,13^. In this study, the samples that tested negative for the parasites in nPCR and were verified by microscopic evaluation were used as true negatives for determining the CV and LOD. When the parasitaemia percentage determined by our automated system was higher than the CV, the sample was considered as parasite-positive. Conversely, when the parasitaemia percentage determined by the system was lower than the CV, the sample was considered as parasite-negative.

The significance of discordance was measured using the Welch’s t-test, Chi-squared test or McNemar’s test. All statistical analyses were performed using R version 3.6.0. Exact 95% CI were computed for sensitivity, specificity, positive and negative predictive values, and accuracy using binomial distributions with the Clopper-Pearson method^38^. Pearson’s correlation test and Spearman’s rank-correlation test were used to evaluate the degree of correlation between the percentage parasitaemia obtained by our diagnostic system and that obtained by microscopy. A linear regression analysis was also performed to correlate the parasitaemia values obtained by these two detection methods^39^. The difference was considered statistically significant when the P-value was less than 0.05.

## Supplementary information

Supplemental manuscript.

Dataset 1.

Dataset 2.

Dataset 3.

Dataset 4.

Dataset 5.

## Data Availability

All data generated or analysed during this study are included in this manuscript (and its Supplementary Information files).
