# Supplementary material for: Development of a quantitative, portable, and automated fluorescent blue-ray device-based malaria diagnostic equipment with an on-disc SiO2 nanofiber filter
Source: Sci Rep. 2020 Apr 20;10:6585. doi: 10.1038/s41598-020-63615-2 (PMC7171072; doi:10.1038/s41598-020-63615-2)
Supplement: Supplementary file 1 — Supplemental manuscript. [file 41598_2020_63615_MOESM1_ESM.docx]

Supplementary Information

Title:

Development of a quantitative, portable, and automated fluorescent blue-ray device-based malaria diagnostic equipment with an on-disc SiO_2_ nanofiber filter

Full author list:

Takeki Yamamoto^a, b^, Muneaki Hashimoto^c^*, Kenji Nagatomi^b^, Takahiro Nogami^b^, Yasuyuki Sofue^b^, Takuya Hayashi^b^, Yusuke Ido^c^, Shouki Yatsushiro^c^, Kaori Abe^c^, Kazuaki Kajimoto^c^, Noriko Tamari^d^, Beatrice Awuor^e^, George Sonye^e^, James Kongere^f^, Stephen Munga^g^, Jun Ohashi^h^, Hiroaki Oka^b^, Noboru Minakawa^d^, Masatoshi Kataoka^c^, Toshihiro Mita^a^*

^a^ Department of Tropical Medicine and Parasitology, Faculty of Medicine, Juntendo University

2-1-1 Hongo, Bunkyo-ku, Tokyo, 113-8421, Japan

^b^ Panasonic Corporation, Automotive & Industrial Systems Company, Kadoma 1006, Kadoma, Osaka, 571-8506, Japan

^c^ National Institute of Advanced Industrial Science and Technology (AIST), Health Research Institute, 2217-14 Hayashi-cho, Takamatsu, Kagawa 761-0395 Japan

^d^ Nagasaki University, Institute of Tropical Medicine, 1-12-4 Sakamoto, 852-8523, Nagasaki, Japan

^e^ Ability to Solve by Knowledge Project, P.O. Box 30, 40305, Mbita, Homa Bay, Kenya

^f^ Nagasaki University Nairobi Research Station, NUITM-KEMRI Project, P.O. Box 19993, 00202, Nairobi, Kenya

^g^ Centre for Global Health Research, Kenya Medical Research Institute, P.O. Box 1578, 40100, Kisumu, Kenya

^h^ Department of Biological Sciences, Graduate School of Science, The University of Tokyo, 7-3-1 Hongo, Bunkyo-ku, Tokyo, 113-8654, Japan

* Correspondence to:

Toshihiro Mita ([tmita@juntendo.ac.jp](mailto:tmita@juntendo.ac.jp)), Muneaki Hashimoto ([muneaki-hashimoto@aist.go.jp](mailto:muneaki-hashimoto@aist.go.jp))


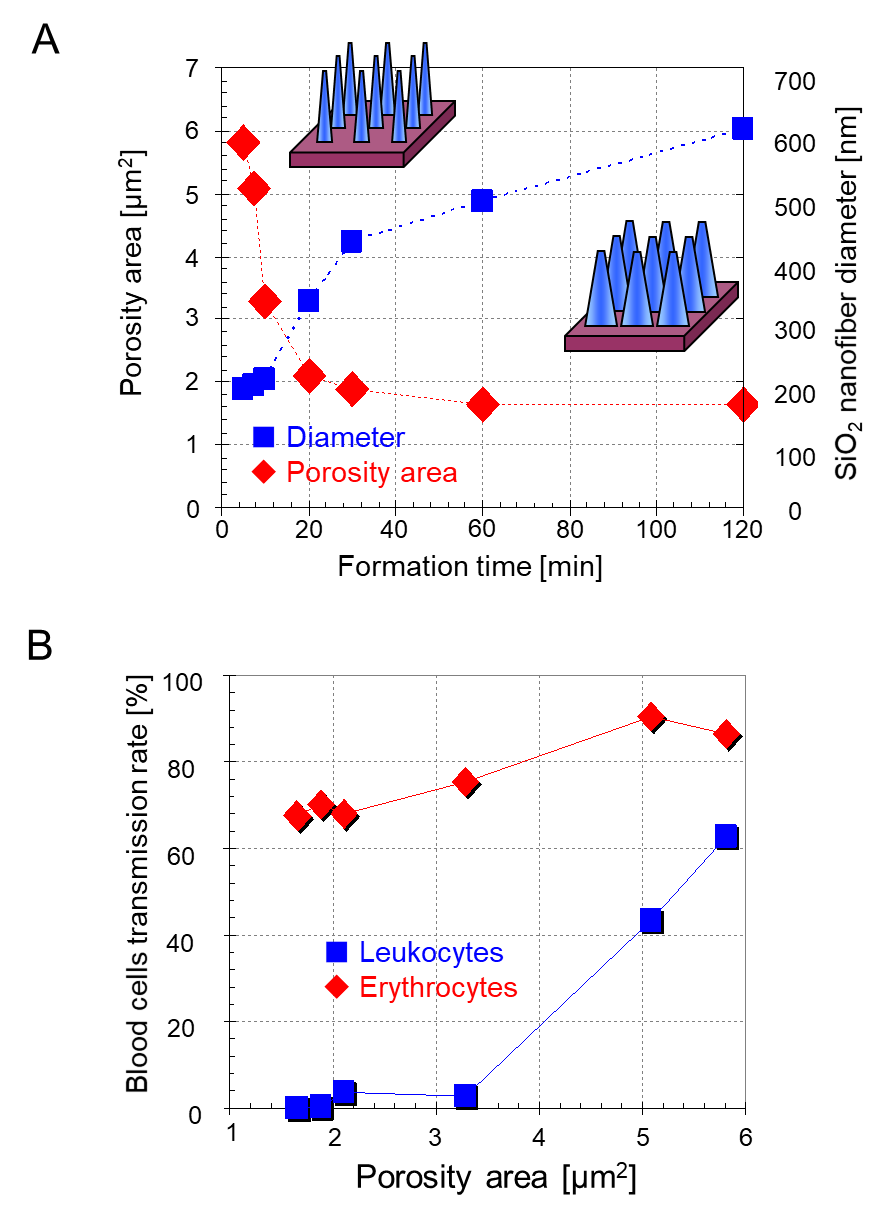


**Supplementary Fig. S1.** SiO_2_ nanofiber formation conditions and blood cell passage performance. (A) Relationship between SiO_2_ nanofiber formation time and selective porosity area. (B) Relationship between selective porosity area and transmission rate of leukocytes and erythrocytes.


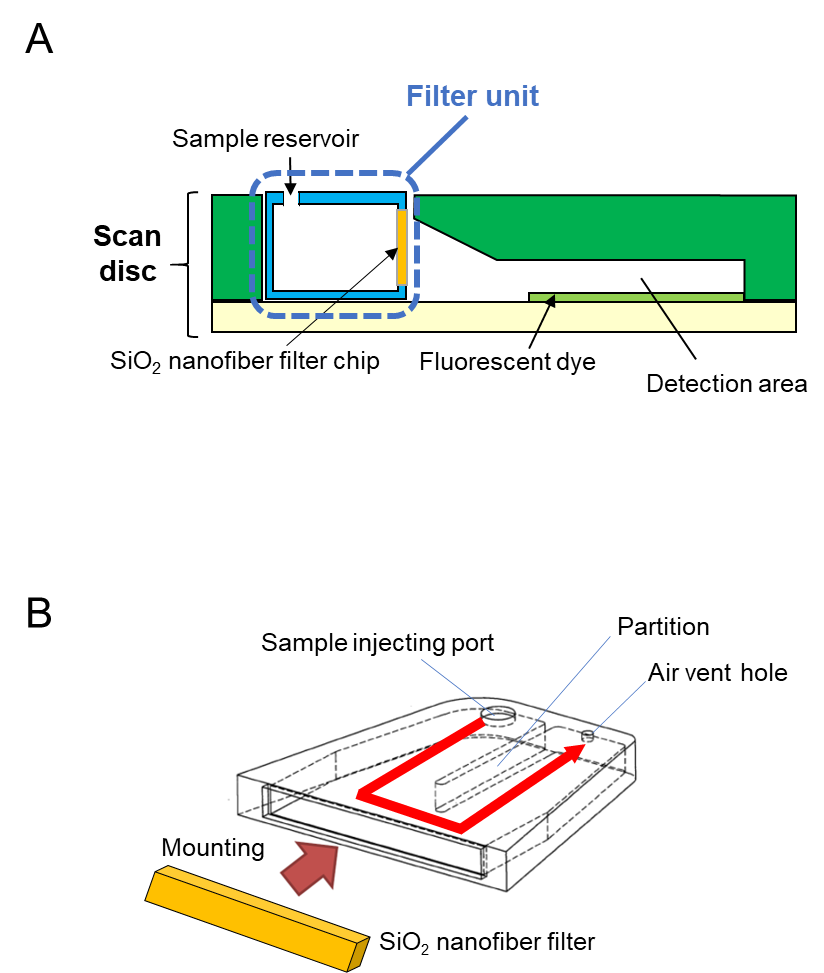


**Supplementary Fig. S2.** (A) Schematic diagram of a cross section of the scan disc and (B) Details of the filter unit design


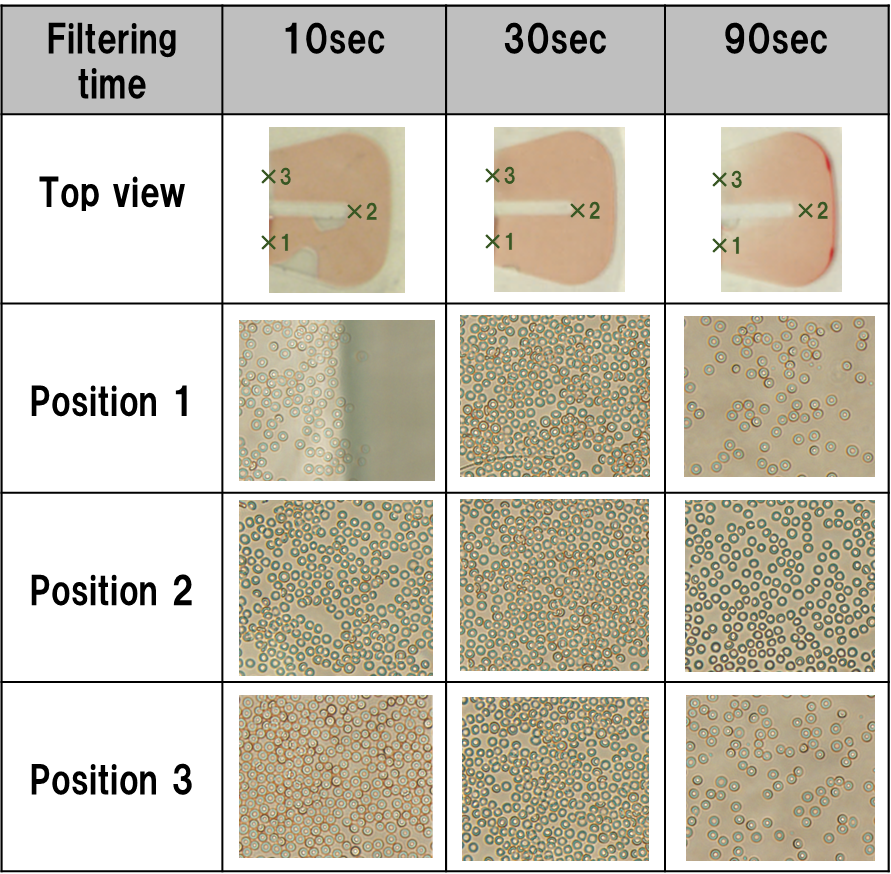


**Supplementary Fig. S3****.** Optimization of rotation speed and rotation time of on-disc SiO_2_ nanofiber filter.


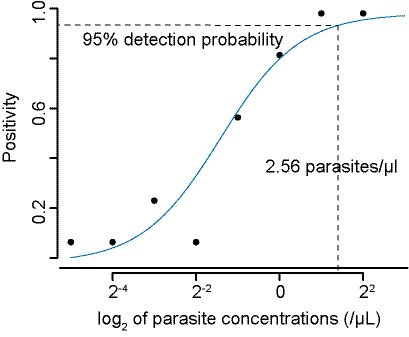


**Supplementary Fig. S4.** Limit of detection of 18S rRNA PCRs. 95% probability of limit of detection of 18S rRNA PCRs. Dashed lines are 95% detection probability of parasite. Continuous line is based on the probit analysis using a serial dilution of 3D7 in vitro culture. A laboratory-adapted 3D7 clone was mixed into leukocytes and platelets-deleted blood from Japanese volunteers to construct a 1% parasitemia blood sample. 100 uL of blood was used for DNA extraction with a final volume of 100 uL. After the measurement of parasite DNA concentration in the DNA solution, it was diluted to make 2-fold diluted samples (0.0375 to 4 parasites/μL). For PCR, we used 1 uL of diluted samples.

**Supplementary Fig. S5.** Flow diagram for the diagnosis of malaria parasites in the participants.

**
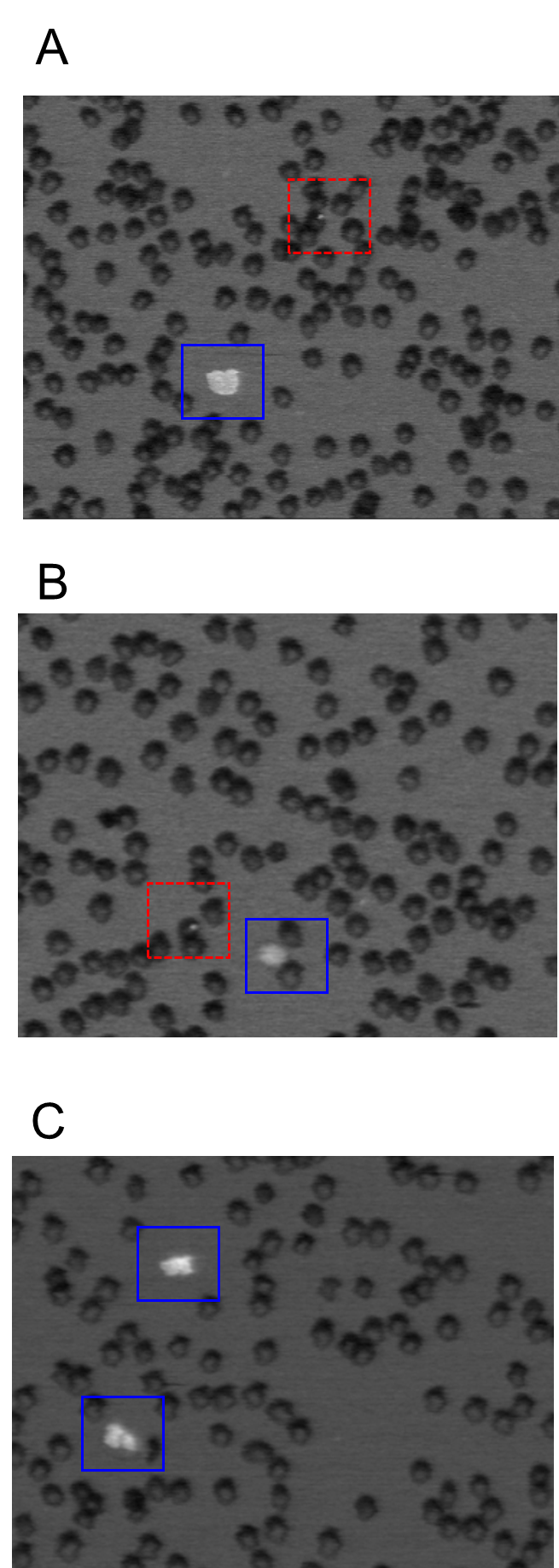
**

**Supplementary Fig. S6**. Fluorescent images of leukocytes (blue-solid line, A, B and C) and malaria-infected erythrocytes (red-dotted line, A and B) on the automated malaria diagnostic system.


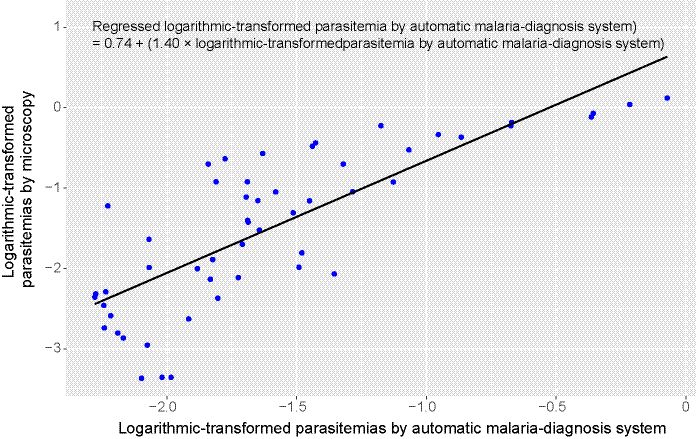


**Supplementary Fig. S7**. Linear regression analysis of the correlation of the percentage parasitemia obtained by the automated malaria diagnostic system with that obtained microscopically.


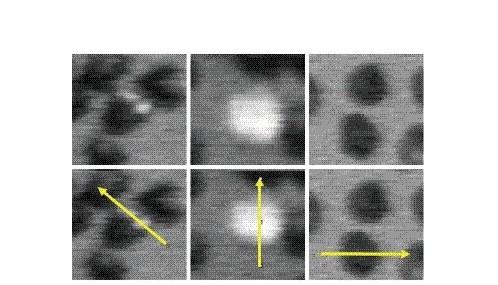


**Supplementary Fig. S8**. Direction of the fluorescence-intensity measurements for malaria, leukocytes and erythrocytes.

**Supplementary Table S2.** Critical value (CV) and limit of detection (LOD) of parasites with automated malaria diagnostic system

**Supplementary Table S3.** Diagnostic accuracy of automated malaria diagnostic system for 40 Japanese volunteers

**Supplementary Table S4.** Degree of coincidence between parasitemias obtained by automated malaria diagnostic system and microscopy
